# Normalizing junk food: The frequency and reach of posts related to food and beverage brands on social media

**DOI:** 10.1371/journal.pdig.0000630

**Published:** 2024-10-31

**Authors:** Monique Potvin Kent, Meghan Pritchard, Christine Mulligan, Lauren Remedios

**Affiliations:** 1 School of Epidemiology and Public Health, Faculty of Medicine, University of Ottawa, Ottawa, Ontario, Canada; 2 Interdisciplinary School of Health Sciences, Faculty of Health Sciences, University of Ottawa, Ottawa, Ontario, Canada; University of Sao Paulo: Universidade de Sao Paulo, BRAZIL

## Abstract

Food and beverage marketing on social media contributes to poor diet quality and health outcomes for youth, given their vulnerability to marketing’s effects and frequent use of social media. This study benchmarked the reach and frequency of earned and paid media posts, an understudied social media marketing strategy, of food brands frequently targeting Canadian youth. The 40 food brands with the highest brand shares in Canada between 2015 and 2020 from frequently marketed food categories were determined using Euromonitor data. Digital media engagement data from 2020 were licensed from Brandwatch, a social intelligence platform, to analyze the frequency and reach of brand-related posts on Twitter, Reddit, Tumblr, and YouTube. The 40 food brands were mentioned on Twitter, Reddit, Tumblr, and YouTube a total of 16.85M times, reaching an estimated 42.24B users in 2020. The food categories with the most posts and reach were fast food restaurants (60.5% of posts, 58.1% of total reach) and sugar sweetened beverages (29.3% of posts, 37.9% of total reach). More men mentioned (2.77M posts) and were reached (6.88B users) by the food brands compared to women (2.47M posts, 5.51B users reached). The food and beverage brands (anonymized), with the most posts were fast food restaurant 2 (26.5% of the total posts), soft drink 2 (10.4% of the total posts), and fast food restaurant 6 (10.1% of the total posts). In terms of reach, the top brands were fast food restaurant 2 (33.1% of the total reach), soft drink 1 (18.1% of the total reach), and fast food restaurant 6 (12.2% of the total reach). There is a high number of posts on social media related to food and beverage brands that are popular among children and adolescents, primarily for unhealthy food categories and certain brands. The conversations online surrounding these brands contribute to the normalization of unhealthy food and beverage intake. Given the popularity of social media use amongst of children and adolescents, policies aiming to protect these vulnerable groups need to include the digital food environment.

## Author summary

Digital media, including social media, is emerging as a top source of youth’s exposure to food marketing for products that are energy-dense, and nutrient-poor. Utilizing earned and paid media is one strategy that brands can use to engage with consumers and promote their products. We studied how often top Canadian food and beverage brands are being mentioned by users on social media, and the reach these posts have amongst users. Our study highlighted that there is a large volume of posts on social media related to food and beverage brands. The 40 included brands were mentioned over 16 million times by users, reaching an estimated 42 billion total users within a one-year period. Most posts were related to less healthy food categories and certain brands, which could be contributing to the normalization of unhealthy food and beverage intake by youth. Given the popularity of social media use amongst youth, this study supports the need for policies to protect this vulnerable group in the digital food environment.

## 1.0 Introduction

Obesity is a global health concern for people of all ages [1]. Food environments, which include the social, physical, economic, and policy contexts that impact dietary intake [2] are recognized as a determinant of obesity and other diet-related health issues [3]. Food and beverage marketing is a particularly negative contributor to the food environment, as most commercial advertising promotes foods high in sugar, fat, and sodium, and reinforces consumer consumption of such products [4–7].

Children and adolescents are particularly vulnerable to food marketing and numerous studies have shown that increased exposure to unhealthy (i.e., high in sugars, sodium and fats) food and beverage advertisements negatively impacts their food preferences, diets and weight [8–10]. Though traditionally, children and adolescents have been extensively targeted by food marketing on television, increased screen time among youth and the proliferation of digital devices has led food and beverage companies to re-focus their marketing efforts to digital platforms, including social media. Social media platforms encourage interactivity and engagement with branded content and enable food and beverage companies to appeal to and target demographic segments and individuals in a way that traditional media, such as television, does not permit [3].

On social media, users can be exposed to food marketing directly by food and beverage companies through owned media (e.g. a company created and owned website, application or social media presence) and paid media (i.e. when a company pays for an ad, like a banner or pop-up ad, to be posted on social media or other digital platform or sponsors a social media influencer to promote a product), or indirectly through earned media (e.g. through word of mouth, customer reviews and social media posts generated by individual users that the company has “earned”.) [11–13]. Both earned and paid media, which we will also refer to as “posts” in this study, contribute to the reach and influence of food marketing on social media platforms [12].

Research from Canada examining child and adolescent exposure to food and beverage products on social media found that 18% of all exposures were posts generated by users other than the companies themselves, and that among the content that featured food and beverage brands or products, 96% of these were excessive in either sodium, fats, or sugar [14]. Food and beverage products shared by friends or family members on social media can have a stronger impact on youth than company branded posts due to increased trust in users’ social networks [15]. This indicates that such posts can play a significant role in shaping youth’s food choices and behaviours, making it crucial to understand its impact on young audiences. Earned and paid media may also to “blur the lines” between advertising and social media content, and marketers can use this to their advantage to engage consumers and encourage them to share branded content through the use of hashtags, competitions, and other interactive activities on social media [3,14,15]. For example, one particularly successful example of earned media was a campaign by Coca-Cola called “Share a Coke” which involved printing common names on the labels of coke bottles and encouraging consumers to share the hashtag “#ShareaCoke” [16]. According to industry reports, this led to an increase of 1.25 million teen users trying Coca-Cola in the summer that followed the campaign [16]. The sharing of branded content can stimulate and reinforce dietary preferences and behaviours driven by indirect food and beverage marketing occurring via content shared by individual users. This is particularly concerning because youth are large consumers of digital media [17,18]. In Canada, 36% of children aged 10–13 years old spend more than 3 hours a day consuming digital media [19].

A comprehensive understanding of the scope of posts related to food and beverage brands is necessary to inform future digital marketing policies aiming to protect children from its negative impacts. To our knowledge, no study has benchmarked posts on popular social media websites surrounding food and beverage brands popular with children and adolescents. This study will provide a detailed examination of posts surrounding popular food and beverage brands on social media by analyzing the frequency and reach of social media posts for food brands frequently targeted at children in Canada.

## 2.0 Methods

### 2.1 Brand selection

The top 40 food brands by sales in Canada were first identified. To do this, the top four food and beverage categories that children and adolescents were most exposed to on social media apps were identified based on previous research [14]. Food categories that children and adolescents were most exposed to were the focus as youth are very vulnerable to food and beverage marketing and spend a large number of hours on digital devices [19]. These four categories included fast food restaurants, sugar-sweetened beverages, candy & chocolate, and snacks. The top 10 brands by sales in Canada in similar food categories were then drawn from Euromonitor, a company that provides market research reports and statistics on products, industries, demographics, and lifestyle trends [20]. Data on brand shares (proportion of sales within a market) from 2015–2020 for the four food categories were examined in Euromonitor, and the top 10 brands within each food category were selected. Table 1 lists the selected food categories used for the purposes of this study, and their corresponding Euromonitor categories. As sugar sweetened beverages comprised 5 separate categories in the Euromonitor data (including soft drinks–carbonates; soft drinks–juices; soft drinks–energy drinks; soft drinks–sports drinks; soft drinks–ready-to-drink teas) two brands from each subcategory were selected. To prevent identifying the brands by name, the top 10 brands in each category were numbered from 1 to 10, with the exception of the sugar sweetened beverages (which were numbered either 1 or 2 in each subcategory) and were therein referred to as their food category and corresponding number between 1 and 10 (e.g., fast food restaurant 1, snack 4).

**Table 1:** Food and beverage categories.

Food/Beverage Category	Euromonitor Category
Fast food restaurants	Limited-service restaurants
Sugar sweetened beverages Soft drinks Fruit drinks Energy drinks Sports drinks Teas	Soft drinks–carbonates Soft drinks–juices Soft Drinks–energy drinks Soft drinks–sports drinks Soft drinks–RTD (ready-to-drink) teas
Candy & chocolate	Confectionary
Snacks	Savoury Snacks

### 2.2 Brandwatch and Data Collection

Data on digital media engagement from 2020 were licensed from Brandwatch, a social intelligence platform that allows users to analyze and track “conversations” (i.e., social media posts) occurring online on various digital platforms [21]. These platforms include news sources, blogs, forums, reviews, and social media platforms (including Facebook, Instagram, Twitter, YouTube, Tumblr, and Reddit). With online conversation as the focus of this research, only social media platforms were analyzed. Due to limitations in Brandwatch’s access to data on certain social media sites, only data from Twitter, Tumblr, YouTube, and Reddit were used. Brandwatch has varying levels of access to data depending on the social media platform. See S1 Appendix for a complete description of Brandwatch’s coverage and access by platform.

Data on posts regarding the top 40 brands were collected on Brandwatch through the use of “queries”. A query is Brandwatch’s equivalent of a search engine; it is a string of key words stitched together using operators (OR, AND, NOT, etc.), which allows Brandwatch to search social media and find posts which include these key words. This may include text only posts as well as picture posts with text captions. Spelling variations and colloquial terms for the 40 brands were included in search queries in order to capture as much of the online conversation as possible. Colloquial terms included common names used for brands, such as “Tim’s” for Tim Hortons. Any posts containing posts of the 40 brands in 2020 (January 1^st^ to December 31^st^, 2020) were captured and analyzed. To restrict our data to Canadian data, Brandwatch’s country location codes were included in the queries. By including these country codes, posts that were “geotagged” (geographically tagged posts as a result of users identifying their location) in countries other than Canada were excluded. While our methodology captured all posts, we were not able to capture posts by age group (i.e. posts specifically from children or adolescents) due to the limitations of the Brandwatch data. Furthermore, only posts authored by individual users (excluding those directly from companies) were included. This approach captured earned media and certain types of paid media, such as influencer or celebrity posts, however it is worth noting that this approach did not cover all types of earned and paid media.

The following key outcome measures were drawn from Brandwatch for all included platforms: the frequency of posts (i.e., a measure of the number of times a brand was mentioned on the examined social media sites), the net sentiment (i.e., a measure to determine the sentiment regarding a brand that is calculated by subtracting the number of negative posts from the positive posts and dividing by the sum of the negative and positive posts. It is expressed as a value between -5 to 5. A sentiment of 5 indicates a more positive opinion whereas a sentiment of -5 indicates a more negative opinion), and the aggregate reach (i.e., an estimate of the number of total individuals that have viewed each post/mention, not unique users). Exclusively on Twitter, we examined the number of impressions (i.e., an estimate of the number of times a Tweet has been viewed that is calculated by summing the author’s and Retweeter’s followers), the total followers (i.e., the sum of all of the followers of those that have Tweeted about a given brand).

For all measures, gendered data were collected when available. Brandwatch collects binary gender (i.e., man/woman) data for posts when algorithms can determine gender from keywords on users’ profiles. All data was compiled in an Excel spreadsheet and organized by food category.

## 3.0 Results

### 3.1 Frequency of brand posts and aggregate reach

Overall, the 40 food and beverage brands examined were mentioned on Twitter, Reddit, Tumblr, and YouTube a total of 16.85M times, reaching an estimated 42.24B users in 2020. The food categories with the most posts and reach were fast food restaurants (60.5% of the posts, 58.1% of total reach), followed by sugar sweetened beverages (29.3% of the posts, 37.9% of total reach) (Table 2). Snacks and candy & chocolate food categories were mentioned considerably less than the other two food categories (4.6% and 5.6% of total posts, respectively, and 1.6% and 2.4% of total reach, respectively). More men mentioned and were reached by the 40 food brands, with a total of 2.77M posts and a reach of 6.88B for men, compared to a total of 2.47M posts and a reach of 5.51B for women. This was true for both posts and reach across all food categories, except candy & chocolate, which had more posts from women (407,194 posts) compared to men (162,479 posts).

**Table 2:** Frequency of total posts and total reach, by gender, for the top 40 brands marketed to children*, by food category on Twitter, Reddit, Tumblr, and YouTube in 2020.

	Posts n (%)	Posts—men n (%)	Posts—women n (%)	Reach n (%)	Reach—men n (%)	Reach—women n (%)
Fast food (FF) restaurants	10.2M* **(60.5%)**	**1.72M (62.1%)**	**1.39M (56.2%)**	24.53B* (58.1%)	**4.05B (58.9%)**	**3.19B (57.9%)**
FF restaurant 1	424,187 (2.5%)	109,195 (3.9%)	84,627 (3.4%)	940.33M (2.2%)	566.74M (8.2%)	90.96M (1.7%)
FF restaurant 2	4.46M (26.5%)	719,470 (26.0%)	584,028 (23.6%)	13,99B (33.1%)	1.71B (24.9%)	1.45B (26.3%)
FF restaurant 3	75,404 (0.4%)	13,198 (0.5%)	10,672 (0.4%)	66.45M (0.2%)	15.18M (0.2%)	10.01M (0.2%)
FF restaurant 4	364,902 (2.2%)	54,247 (2.0%)	58,382 (2.4%)	277.59M (0.7%)	33.46M (0.5%)	71.16M (1.3%)
FF restaurant 5	1.52M (9.0%)	232,371 (8.4%)	179,279 (7.3%)	2.4B (5.7%)	384,264,520 (5.6%)	372.45M (6.8%)
FF restaurant 6	1.69M (10.1%)	304,272 (11.0%)	242,195 (9.8%)	5.17B (12.2%)	1,024,296,644 (14.9%)	953.96M (17.3%)
FF restaurant 7	50,972 (0.3%)	9,034 (0.3%)	5,828 (0.2%)	54.03M (0.1%)	12,643,464 (0.2%)	6.77M (0.1%)
FF restaurant 8	219,116 (1.3%)	41,103 (1.5%)	40,107 (1.6%)	195.15M (0.5%)	41,034,212 (0.6%)	35.24M (0.6%)
FF restaurant 9	99,299 (0.6%)	13,293 (0.5%)	9,168 (0.4%)	75.52M (0.2%)	12,176,679 (0.2%)	8.44M (0.2%)
FF restaurant 10	1.28M (7.6%)	220,781 (8.0%)	173,549 (7.0%)	1.36B (3.2%)	250,384,350 (3.6%)	192.06M (3.5%)
Sugar Sweetened beverages	**4.94M (29.3%)**	**759,025 (27.4%)**	**563,369 (22.8%)**	**16.02B (37.9%)**	**2.51B (36.5%)**	**2.07B(37.6%)**
Soft drink 1	1.17M (7.0%)	170,819 (6.2%)	137,067 (5.5%)	7.66B(18.1%)	769.79M (11.2%)	1.45B(26.3%)
Soft drink 2	1.75M (10.4%)	245,758 (8.9%)	235,613 (9.5%)	2.47B (5.8%)	368.58M (5.4%)	226.29M (4.1%)
Sport drink 1	524,973 (3.1%)	108,758 (3.9%)	59,306 (2.4%)	4.32B(10.2%)	1.08B (15.7%)	259,310,912 (4.7%)
Sport drink 2	63,426 (0.4%)	9,191 (0.3%)	6,189 (0.3%)	41.21M (0.1%)	7.36M (0.1%)	5.63M (0.1%)
Fruit juice 1	11,907 (0.1%)	729 (0.0%)	564 (0.0%)	144,26M (0.3%)	1.18M (0.0%)	380,529 (0.0%)
Fruit juice 2	1,032 (0.0%)	126 (0.0%)	96 (0.0%)	824,146 (0.0%)	244,527 (0.0%)	104,543 (0.0%)
Energy drink 1	217,163 (1.3%)	24,434 (0.9%)	16,562 (0.7%)	151.06M (0.4%)	21.68M (0.3%)	21.68M (0.4%)
Energy drink 2	1.18M (7.0%)	197,370 (7.1%)	105,682 (4.3%)	1.22B(2.9%)	261.22M (3.8%)	108.26M (2.0%)
Tea 1	8,443 (0.1%)	1,168 (0.0%)	1,034 (0.0%)	6.04M (0.0%)	1.18M (0.0%)	770,418 (0.0%)
Tea 2	5,873 (0.0%)	672 (0.0%)	1,256 (0.1%)	5.5M (0.0%)	617,813 (0.0%)	570,679 (0.0%)
Snacks	**773,279 (4.6%)**	**128,317 (4.6%)**	**113,152 (4.6%)**	**676.19M (1.6%)**	**121,15M (1.8%)**	**113.69M (2.1%)**
Snack 1	46,150 (0.3%)	6,163 (0.2%)	6,070 (0.2%)	40.58M (0.1%)	7.31M (0.1%)	6.09M (0.1%)
Snack 2	610,596 (3.6%)	100,066 (3.6%)	87,820 (3.6%)	475.15M (1.1%)	87.77M (1.3%)	90.2M (1.6%)
Snack 3	9,392 (0.1%)	1,213 (0.0%)	1,095 (0.0%)	37.17M (0.1%)	1.75M (0.0%)	1.28M (0.0%)
Snack 4	7,113 (0.0%)	1,646 (0.1%)	1,688 (0.1%)	10.21M (0.0%)	2.2M (0.0%)	1.46M (0.0%)
Snack 5	30,341 (0.2%)	6,097 (0.2%)	6,132 (0.2%)	43.23M (0.1%)	5.34M (0.1%)	4.0M (0.1%)
Snack 6	2,213 (0.0%)	401 (0.0%)	176 (0.0%)	3.56M (0.0%)	505.637 (0.0%)	141,519 (0.0%)
Snack 7	27,065 (0.2%)	5,596 (0.2%)	3,989 (0.2%)	28.56M (0.1%)	9.51M(0.1%)	4.04M (0.1%)
Snack 8	32,377 (0.2%)	5,251 (0.2%)	5,534 (0.2%)	31.7M (0.1%)	5.54M (0.1%)	5.88M (0.1%)
Snack 9	7,847 (0.0%)	1,859 (0.1%)	608 (0.0%)	5.91M (0.0%)	1.15M (0.0%)	586,716 (0.0%)
Snack 10	185 (0.0%)	25 (0.0%)	40 (0.0%)	99,127 (0.0%)	50,659 (0.0%)	16,036 (0.0%)
Candy & Chocolate	**947,446 (5.6%)**	**162,479 (5.9%)**	**407,194 (16.5%)**	**1.01B(2.4%)**	**193.7M (2.8%)**	**135.74M (2.5%)**
Candy/chocolate 1	188,235 (1.1%)	28,026 (1.0%)	34,729 (1.4%)	187.25M (0.4%)	37,395 (0.0%)	32,189 (0.0%)
Candy/chocolate 2	96,688 (0.6%)	11,195 (0.4%)	13,479 (0.5%)	164.56M (0.4%)	36.65M (0.5%)	35.36M (0.6%)
Candy/chocolate 3	51,585 (0.3%)	6,909 (0.2%)	11,941 (0.5%)	48.68M (0.1%)	10.14M (0.1%)	10.51M (0.2%)
Candy/chocolate 4	297,760 (1.8%)	42,228 (1.5%)	44,050 (1.8%)	291.82M (0.7%)	49.35M (0.7%)	44.83M (0.8%)
Candy/chocolate 5	103,929 (0.6%)	36,528 (1.3%)	5,935 (0.2%)	113.22M(0.3%)	52.47M (0.8%)	8.07M (0.1%)
Candy/chocolate 6	2,272 (0.0%)	499 (0.0%)	566 (0.0%)	3.22M (0.0%)	864,470 (0.0%)	905,229 (0.0%)
Candy/chocolate 7	3,358 (0.0%)	743 (0.0%)	510 (0.0%)	8.52M (0.0%)	3.07M (0.0%)	215,147 (0.0%)
Candy/chocolate 8	178,068 (1.1%)	29,836 (1.1%)	290,848 (11.8%)	163.59M (0.4%)	30.5M (0.4%)	28.61M (0.5%)
Candy/chocolate 9	15,988 (0.1%)	4,235 (0.2%)	3,475 (0.1%)	23.28M (0.1%)	7.51M (0.1%)	5.33M (0.1%)
Candy/chocolate 10	9,563 (0.1%)	2,280 (0.1%)	1,661 (0.1%)	9.69M (0.0%)	3.11M (0.0%)	1,88M (0.0%)
Total	**16.85M (100.0%)**	**2.77M** **(100.0%)**	**2.47M** **(100.0%)**	**42.24B** **(100.0%)**	**6.88B** **(100.0%)**	**5.51B** **(100.0%)**

Source: Brandwatch, 2020; *Top 40 brands with the highest brands shares in Canada between 2015 and 2020 from the food categories that children and adolescents were most exposed to on social media in Potvin Kent et al. (2019).

*M refers to million and B refers to billion

In terms of food and beverage brands, those with the most posts were fast food restaurant 2 (26.5% of the total posts), soft drink 2 (10.4% of the total posts), fast food restaurant 6 (10.1% of the total posts) (Table 2). These three brands alone made up 47% of the total posts. In terms of reach, the top brands were fast food restaurant 2 (33.1% of the total reach), soft drink 1 (18.1% of the total reach), and fast food restaurant 6 (12.2% of the total reach). These three brands made up 63.4% of the total reach. When considering gender differences on the individual brand level, almost twice as many men mentioned energy drink 2 (197,370 (7.1%) posts) compared to women (105,682 (4.3%) posts) and candy and chocolate 8 had considerably more posts by women (290,848 (11.8%)) compared to men (29,836 (1.1%) posts). In terms of reach, more women (1.45B (26.3%)) were reached by soft drink 1 compared to men (769.79M (11.2%)), and more men (1.08B (15.7%)) were reached by sport drink 1 compared to women (259.31M (4.7%)).

### 3.2 Net sentiment

Most food categories were positively rated both overall and among both genders, with the greatest net sentiment observed for candy & chocolate (1.49). An exception was for fast food restaurants which had an overall net sentiment of -0.78 (Table 3). The greatest differences between men and women were observed for candy & chocolate, which were rated more positively by women (2.17) compared to men (1.53).

**Table 3:** Average net sentiment scores, by gender, of the top 40 brands marketed to children* by food category on Twitter, Reddit, Tumblr, and YouTube in 2020.

	Net Sentiment	Net Sentiment- Men	Net Sentiment- Women
Fast food (FF) restaurants	**-0.78**	**-0.64**	**-0.51**
FF restaurant 1	-0.18	0.52	0.47
FF restaurant 2	-1.30	-1.24	-1.19
FF restaurant 3	0.53	1.01	1.50
FF restaurant 4	-1.42	-1.13	-1.94
FF restaurant 5	0.36	0.45	0.71
FF restaurant 6	-0.63	-0.88	-0.49
FF restaurant 7	-0.38	-0.63	-0.20
FF restaurant 8	-1.53	-1.86	-1.22
FF restaurant 9	-1.11	-0.48	0.09
FF restaurant 10	-2.12	-2.20	-2.79
Sugar Sweetened beverages	**0.49**	**0.93**	**1.11**
Soft drink 1	-0.10	0.32	0.79
Soft drink 2	0.64	0.58	0.63
Sport drink 1	-0.31	0.49	-0.15
Sport drink 2	-0.19	0.53	0.66
Fruit juice 1	0.31	0.50	0.73
Fruit juice 2	0.82	0.94	2.60
Energy drink 1	-0.99	-0.10	-0.10
Energy drink 2	0.39	1.36	1.15
Tea 1	1.60	1.59	2.03
Tea 2	2.71	3.07	2.76
Snacks	**0.51**	**1.03**	**1.05**
Snack 1	0.59	0.95	1.39
Snack 2	-0.18	0.18	0.06
Snack 3	1.49	2.15	2.00
Snack 4	1.06	1.06	1.27
Snack 5	1.18	1.01	2.88
Snack 6	-0.21	0.52	0.84
Snack 7	2.18	2.67	3.18
Snack 8	0.96	0.87	1.43
Snack 9	0.92	1.93	1.22
Snack 10	-2.85	-1.00	-3.82
Candy & Chocolate	**1.49**	**1.53**	**2.17**
Candy/chocolate 1	0.64	0.93	1.52
Candy/chocolate 2	0.66	0.60	1.89
Candy/chocolate 3	1.47	1.61	2.19
Candy/chocolate 4	0.87	0.71	1.42
Candy/chocolate 5	0.30	0.61	2.00
Candy/chocolate 6	0.82	0.53	1.52
Candy/chocolate 7	4.67	4.39	4.64
Candy/chocolate 8	0.56	0.86	0.33
Candy/chocolate 9	3.06	3.04	3.38
Candy/chocolate 10	1.84	2.04	2.77

Source: Brandwatch, 2020;

*Top 40 brands with the highest brands shares in Canada between 2015 and 2020 from the food categories that children and adolescents were most exposed to on social media in Potvin Kent et al. (2019).

The food and beverage brands with the most positive posts were candy & chocolate 7 (4.67), candy & chocolate 9 (3.06), and tea 2 (2.71). The brands with the most negative posts were snack 10 (-2.85), fast food restaurant 10 (-2.12), and fast food restaurant 8 (-1.53). The top three greatest differences between men and women included fruit juice 3, which scored more positively among women (2.60) compared to men (0.94); snack 10, which scored more negatively among women (-3.82) compared to men (-1.00); and candy & chocolate 5, which scored more positively among women (2.00) compared to men (0.61).

### 3.3 Twitter Followers and Impressions

Overall, the cumulative total of all followers of users who Tweeted about the 40 brands was 383,84B users, and these tweets were seen an estimated 491.21B times (Table 4). Fast food restaurants and sugar sweetened beverages accounted for 98.5% of the followers and 98.2% of the impressions combined (84.7% and 13.7% of the followers and 81.9% of 16.3% of the impressions, respectively). In terms of gender differences, overall, men had more followers and impressions compared to women, with men having 14.71B total followers and 29.15B total impressions compared to women, with only 3.73B total followers and 14.38B total impressions. This was especially true of fast food restaurants as a food category, with this category having 11,07B (75.3%) followers and 20,55B (70.5%) impressions for men compared to 1.98B (53.1%) followers and 9.13B (63.5%) impressions for women.

**Table 4:** Total followers of Twitter users who have Tweeted about the top 40 brands marketing to children*, and Twitter impressions of each mention by food category in 2020.

	Total Followers n (%)	Total Followers- Men n (%)	Total Followers- Women n (%)	Total Impressions n (%)	Total Impressions -Men n (%)	Total Impressions- Women n (%)
Fast food (FF) restaurants	325,03B* **(84.7%)**	**11.07B (75.3%)**	**1.98B** **(53.1%)**	**402.19B** **(81.9%)**	**20.58B** **(70.5%)**	**9.13B** **(63.5%)**
FF restaurant 1	9.65B (2.5%)	7.92B (53.8%)	125.29M* (3.4%)	11.51B (2.3%)	8.32B (28.5%)	398.33M (2.8%)
FF restaurant 2	293.79B (76.5%)	1.25B (8.5%)	863.43M (23.2%)	352.79B (71.8%)	7.19B (24.7%)	5.59B (38.9%)
FF restaurant 3	153.72M (0.0%)	20.46M (0.1%)	16.52M (0.4%)	240.01B (0.0%)	45.94M (0.2%)	26.6M (0.2%)
FF restaurant 4	504.76M (0.1%)	504.76M (3.4%)	53.87M (1.4%)	924.49M (0.2%)	156.34M (0.5%)	131.89M (0.9%)
FF restaurant 5	5.01B (1.3%)	451.30M (3.1%)	356.14M (9.6%)	8.27B (1.7%)	935.87M (3.2%)	815.79M (5.7%)
FF restaurant 6	9.33B (2.4%)	434.21M (3.0%)	264.71M (7.1%)	17.57B (3.6%)	2.64B (9.0%)	1.47B (10.2%)
FF restaurant 7	69.62M (0.0%)	14.29M (0.1%)	7.17M (0.2%)	141.27M (0.0%)	24.2M (0.1%)	13.61M (0.1%)
FF restaurant 8	681.25M (0.2%)	77.89M (0.5%)	48.95M (1.3%)	1.14B(0.2%)	196.5M (0.7%)	146.88M (1.0%)
FF restaurant 9	740.41M (0.2%)	28.2M (0.2%)	33.21M (0.9%)	1.08B (0.2%)	64.97M (0.2%)	7.1M (0.0%)
FF restaurant 10	5.02B (1.3%)	365,82M (2.5%)	211.25M (5.7%)	8.53B(1.7%)	983.49M (3.4%)	525.33M (3.7%)
Sugar Sweetened beverages	**52.78B** **(13.7%)**	**2.98B** **(20.3%)**	**1.18B** **(31.6%)**	**79.89B** **(16.3%)**	**7.32B** **(25.1%)**	**4.24B** **(29.5%)**
Soft drink 1	7.96B(2.1%)	794.3M (5.4%)	362.25M (9.7%)	13.23B (2.7%)	2.08B (7.1%)	1.04B (7.2%)
Soft drink 2	29.1B (7.6%)	602.82M (4.1%)	421.35M (11.3%)	46.82B (9.5%)	2.93B(10.1%)	2.55B (17.7%)
Sport drink 1	2.33B (0.6%)	174.4M (1.2%)	102.92M (2.8%)	3.44B (0.7%)	376.56M (1.3%)	202.02M (1.4%)
Sport drink 2	152.56M (0.0%)	8.72M (0.1%)	9.1M (0.2%)	268.14M (0.1%)	35.35M (0.1%)	32.47M (0.2%)
Fruit juice 1	1.23B (0.3%)	11.32M (0.1%)	511,136 (0.0%)	1.25B (0.3%)	16.98M (0.1%)	1.13M(0.0%)
Fruit juice 2	1.71M (0.0%)	619,987 (0.0%)	449,778 (0.0%)	2.62M (0.0%)	680,414 (0.0%)	574,885 (0.0%)
Energy drink 1	752.23M (0.2%)	55.01M (0.4%)	21.2M (0.6%)	997.09M (0.2%)	90.54M (0.3%)	31.39M (0.2%)
Energy drink 2	11.22B (2.9%)	1.33B (9.0%)	257.93M (6.9%)	13.82B (2.8%)	1.79B (6.1%)	379.37M (2.6%)
Tea 1	9.22M (0.0%)	2.09M (0.0%)	939,975 (0.0%)	13.01M (0.0%)	3.01M (0.0%)	1.32M (0.0%)
Tea 2	24.07M (0.0%)	1.41M (0.0%)	1.06M(0.0%)	47.0M (0.0%)	1.94M (0.0%)	1.41M (0.0%)
Snacks	**2.04B** **(0.5%)**	**234.14M** **(1.6%)**	**243.91M** **(6.5%)**	**3.74B** **(0.8%)**	**596.25M** **(2.0%)**	**485.9M** **(3.4%)**
Snack 1	96.42M (0.0%)	12.81M (0.1%)	7.6M (0.2%)	161.92M (0.0%)	28.64M (0.1%)	18.38M (0.1%)
Snack 2	1.39B (0.3%)	165,67M (1.1%)	201.58M (5.4%)	2.71B(0.6%)	473.44M (1.6%)	419.27M (2.9%)
Snack 3	116.71M (0.0%)	5.81M (0.0%)	1.73M (0.0%)	228.99M (0.0%)	7.13M (0.0%)	2.94M (0.0%)
Snack 4	40.83M (0.0%)	3.84M(0.0%)	2.33M (0.1%)	48,73M (0.0%)	4.84M (0.0%)	2.83M (0.0%)
Snack 5	138.31M (0.0%)	15.74M (0.1%)	9.84M (0.3%)	175.44M (0.0%)	22.57M (0.1%)	12.61M (0.1%)
Snack 6	7.17M (0.0%)	900,119 (0.0%)	315,308 (0.0%)	10.21M (0.0%)	1.34M (0.0%)	427,957 (0.0%)
Snack 7	95.27M (0.0%)	14.55M (0.1%)	7.06M (0.2%)	147.64M (0.0%)	37.51M(0.1%)	9.83M, (0.1%)
Snack 8	180.5M (0.0%)	12.39M (0.1%)	11.96M (0.3%)	215.31M (0.0%)	16.56M (0.1%)	17.24M (0.1%)
Snack 9	26.8M (0.0%)	2.37M (0.0%)	1.45M (0.0%)	40.19M (0.0%)	4.14M (0.0%)	2.34M (0.0%)
Snack 10	143,841 (0.0%)	58,785 (0.0%)	31,323 (0.0%)	217,004 (0.0%)	84,703 (0.0%)	46,739 (0.0%)
Candy & Chocolate	**3.99B** **(1.0%)**	**424.35M** **(2.9%)**	**326.07M** **(8.7%)**	**5,398,666,995** **(1.1%)**	**679.1M** **(2.3%)**	**526.52M** **(3.7%)**
Candy/chocolate 1	1.15B (0.3%)	110.1M (0.8%)	54.61M (1.5%)	1.43B(0.3%)	145,09M (0.5%)	88.24M (0.6%)
Candy/chocolate 2	487,84M (0.1%)	27.96M (0.2%)	106.82M (2.9%)	643.06M (0.1%)	43.91M (0.2%)	124.12M (0.9%)
Candy/chocolate 3	268.04M (0.1%)	16.18M (0.1%)	18.29M (0.5%)	310.04M (0.0%)	23.72M (0.1%)	26.43M (0.2%)
Candy/chocolate 4	844.3M (0.2%)	79.97M (0.5%)	80.78M (2.2%)	1,3B (0.3%)	150.19M (0.5%)	154.41M (1.1%)
Candy/chocolate 5	428.87M (0.1%)	98.47M (0.7%)	9.88M (0.3%)	645.6M (0.1%)	181.71M (0.6%)	37.24M(0.3%)
Candy/chocolate 6	8.33M (0.0%)	1.28M (0.0%)	1.87M (0.1%)	11,74M (0.0%)	1.87M (0.0%)	2.45M (0.0%)
Candy/chocolate 7	14.16M (0.0%)	7.72M (0.1%)	345,209 (0.0%)	29.84M (0.0%)	9.99M (0.0%)	489,869 (0.0%)
Candy/chocolate 8	734.84M (0.2%)	67.37M(0.5%)	44.17M (1.2%)	960.40M (0.2%)	102.76M (0.4%)	78.47M (0.5%)
Candy/chocolate 9	34.12M (0.0%)	8.86M (0.1%)	6.4M (0.2%)	47.73M (0.0%)	12.47M (0.0%)	10.36M (0.1%)
Candy/chocolate 10	16.36M (0.0%)	5.55M (0.0%)	2.9M (0.1%)	25.48M (0.0%)	8.29M (0.0%)	4.32M (0.0%)
Total	**383.84B (100.0%)**	**14.71B (100.0%)**	**3.73B (100.0%)**	**491.21B (100.0%)**	**29.15B (100.0%)**	**14.38B (100.0%)**

Source: Brandwatch, 2020; *Top 40 brands with the highest brands shares in Canada between 2015 and 2020 from the food categories that children and adolescents were most exposed to on social media in Potvin Kent et al. (2019)

*M refers to million and B refers to billion

Fast food restaurant 2 was the most popular brand overall, with 76.5% of all the total followers and 71.8% of the total impressions. This was followed by soft drink 2, with 7.6% of the total followers, and 9.5% of the total impressions. Fast food restaurant 1 was more popular among men, with 7.92B (53.8%) followers and 8.32B (28.5%) impressions for men, compared to only 125.29M (3.4%) followers and 398.33M (2.8%) impressions for women. Fast food restaurant 2 had higher number of followers and impressions for women compared to men, with 863.43M (23.2%) followers and 5.59B (38.9%) impressions for women, and 1.25B (8.5%) followers and 7.19B (24.7%) impressions for men.

## 4.0 Discussion

This study assessed the frequency of posts related to the top 40 brands in 4 food categories frequently advertised to children and adolescents on social media in 2020. The results from this study show 1) that conversations on social media surrounding unhealthy food and beverage brands are ubiquitous, 2) the most commonly discussed brands were fast food restaurants and sugar sweetened beverages, and 3) men are involved in conversations about these brands more frequently than women.

### Prevalence of Conversations About Brands on Social Media

The results from the study show that on Twitter, Reddit, Tumblr, and YouTube, the top 40 brands with the highest brand shares based on food and beverage categories that children and adolescents were most exposed to on social media are being frequently discussed. The 40 brands were mentioned 16.85M times in Canada, reaching 42.24B users in 2020. On Twitter alone, the cumulative followers of all users who mentioned the brands was 383.84B users and these Tweets had an estimated 491.21B impressions. These results indicate that there 40 brands examined are being heavily discussed in posts on social media. The results from our study are consistent with other research. A study conducted in Sweden looking at Instagram posts of 14-year-olds found that 85% of their posts contained food and beverages, and 67.7% of these food and beverage posts were considered to be HFSS foods [22].

The frequency of conversations regarding unhealthy food and beverages on social media is concerning from a public health perspective for a variety of reasons. Children and adolescents are frequent users of social media. In fact, according to the World Health Organization, 78% of children in the UK reported having at least one social media account, such as Facebook or Instagram [23]. In Canada, data from 2015 showed that although they did not meet the age minimum to create an account (i.e., age 13), nearly 1/3 of children in grades 4 to 6 had Facebook accounts [18]. Children are vulnerable to the marketing of unhealthy foods because their cognitive development makes the recognition of all forms of digital marketing challenging [24]. Adolescents are equally vulnerable as they have more financial independence, and their neurocognitive development leads them to be unduly influenced by peers [25]. Secondly, the ubiquitous presence of posts on unhealthy food brands can contribute to the normalization of the consumption of these types of foods [26]. Users sharing brand-related content on social media may cause these unhealthy eating behaviours to become viewed as the norm, and result in exponential user exposure to food-related content with every share, further perpetuating social dietary norms [26,27]. A study on perceived norms on social media showed that when users perceived the consumption of unhealthy foods and beverages to be high based on social media posts, their consumption increased [26].

While this study did not specifically capture children and adolescents’ exposure to earned and paid media on social media, other research in this area demonstrates high rates of exposure to these types of content. One Australian study looked at screen-recordings of youth aged 13–17 years old and found that of all of the food and beverage promotions that they saw, only 16.46% were considered owned media, while 58.77% and 24, 76% were earned and paid media, respectively [13]. Given the prevalence of these types of posts, it is important to consider the potential impact they have in shaping youth dietary behaviours. Digital media plays an increasingly important role in youth’s daily lives which means it is an increasingly important factor to consider in terms of food environments of children and adolescents. Canadian data shows that 32% of 10–13 year old’s and 44% of 14–15 year old’s spend 3+ hours on digital devices per day [17], while other data showed that by the end of high school, 95% of students had a Facebook account while 47% had a Twitter account [18]. Since posts on social media include content shared among peers, youth are particularly vulnerable to this type of influence, as peer influence becomes important during these years of development [27]. While research in this area is limited, there is evidence that companies are actively promoting and encouraging interactions among youth through peer-to-peer networking. A notable example includes Redbull’s #PutACanOnIt campaign, which encouraged users to share creative photos featuring cans of Redbull [28]. In addition, influencer marketing can play an important role in shaping youth dietary behaviors. Influencers, who often have large followings, hold a lot of sway over their followers meaning that they can amplify the reach and impact of food and beverage promotions. One study examining the effect of influencer marketing on children’s snack intake found that children exposed to influencer marketing of an unhealthy snack consumed significantly more of the marketed snack compared to an alternative brand [29]. When considering the high frequency of and nature of posts, the vulnerability of youth to influence in the food environment, and the rates of children and adolescents with social media accounts, there is a clear health concern for Canadian youth. Governments need to consider this environment when developing policy to protect children and adolescents from unhealthy food marketing.

### Fast food restaurant and sugar sweetened beverage are the most popular food categories in posts

The vast majority of the brands discussed on social media came from either the fast-food restaurant category or the sugar sweetened beverage category. Fast food made up 60.5% of the posts, 62.1% of the reach, 84.7% of the followers, and 81.9% of the impressions. Moreover, it appears as though certain brands are particularly prominent within posts in this food category. For instance, fast food restaurant 2 made up almost half of the posts and reach and the vast majority of Twitter followers and impressions. These findings are consistent with other research, which shows that children and adolescents are more frequently exposed to fast food compared to other food categories on social media [14,27]. Fast food remains one of the food categories that is most frequently advertised to youth on different platforms, such as on television [30]. A recent Canadian study on advertising expenditures also indicated that in 2019, fast food advertising had the highest expenditures across television, print, radio and outdoor advertising [31]. The predominance of fast food advertising is worrisome as among individuals of all ages, fast food consumption is associated with poor diet, increased caloric intake, and worse health outcomes [32]. These health outcomes include fat gain, compromised insulin functioning, or increased risk of cardiovascular disease or diabetes [33]. Interestingly, fast food restaurants received an overall negative net sentiment (-0.78), which is surprising given their popularity.

Sugar sweetened beverages were the second most popular food category, with 29.3% of the posts, 37.9% of the reach, 13.7% of the total followers, and 16.3% of the total impressions. This is supported by other research looking at the food and beverage brands that children and adolescents were most exposed to on social media. One Canadian study found that following fast food restaurants (44%), the food category with the second highest marketing exposures was sugar-sweetened beverages (9%) [14]. The consumption of sugary drinks is linked to excess weight or obesity and other health complications such as cardiovascular diseases, diabetes, and dental problems [34]. As frequent consumers, youth are one of the primary target markets for sugary beverage brands and sugar sweetened beverages are one of the main sources of sugar in the diets of adolescents in Canada [35]. The results of our study also showed that select beverage brands made up a large proportion of the total measures, indicating the dominance of certain key brands within this food category. For example, the two soft drink brands accounted for the majority of the conversation surrounding the sugar sweetened beverage brands in all four outcome measures. The frequent discussion of these popular brands on social media is concerning as such discourse may further contribute to the normalization and social acceptance of consuming sugar sweetened beverages.

### Gender differences

The results of the study also show that men had higher posts, reach, followers, and impressions surrounding food and beverage brands compared to women on social media. Research on gender differences and food consumption shows that women are more prone to healthy eating choices compared to men, being more likely to consume fruit and vegetables than men, while men are more likely to consume fast food and soft drinks than women [36]. Other research supports this concept, with ultra-processed foods making up a higher percentage (49.8%) of the diets of men, compared to women (46.7%) [37]. Furthermore, a scoping review on youth’s dietary intakes and food marketing found that the food preferences of boys were more strongly influenced by marketing than girls by exposure to marketing for unhealthy foods and beverages [38]. Additionally, the data suggests that brands might be employing targeted marketing strategies to appeal to specific genders. For instance, women rated candy & chocolate more positively (2.17) compared to men (1.53), while men showed slightly higher net sentiment scores for certain fast food restaurants. This is consistent with other research, which shows that advertisers may utilize gender to their benefit to target users [39,40]. While the current study did not delve into the rationale of these findings, the results suggest clear gender-based differences in posts that warrant further consideration from both a research and a policy perspective.

### Strengths and limitations

To our knowledge, this study is the first to examine the frequency and reach of user-generated social media posts containing posts of food and beverage products over the course of an entire year, on multiple social media platforms. While our study provides unique snapshot of the types of foods and the brands that users are most frequently discussing, and how many users are being exposed to this content, further research is needed to directly link youth interactions with this content and their dietary behaviours. Previous studies indicate that children and adolescents are heavily exposed to and influenced by digital marketing, yet our methodology did not explicitly capture the age demographics of users involved in posts. Thus, our conclusions highlight the potential public health implications of earned and paid media, but call for more targeted studies to confirm these interactions. Other limitations of the study are inherent to the nature of Brandwatch data. Firstly, the queries did not necessarily capture every mention related to the included food and beverage categories. This is because 1) the queries were written in English, so any posts by Canadians in another language (e.g., French) were not included, and 2) there is likely a wide range of spellings and when referring to the 40 included brands, however for methodological consistency, only main variations/spellings were included. These two factors likely lead to an underestimation of the frequency and reach of brands. Secondly, Facebook and Instagram data were unable to be included in our study due to limitations in the data agreement between Brandwatch and Facebook or Instagram. Facebook and Instagram are extremely popular social media platforms, so this study is missing key information related to social media and is likely underestimating the total frequency and reach of posts related to food brands in Canada. Additionally, data on the ages of the users who were involved in the posts was not available, so although we can make inferences based on other evidence regarding child and teen social media use, estimating youth exposure and to these posts was not within the scope of this study. Furthermore, despite the inclusion of country location codes in the data queries, untagged posts from users outside Canada may have been included in the analysis. Additionally, Brandwatch can only collect gendered data for accounts that have disclosed their gender through using keywords (e.g., “father” or “husband” would translate to “man”). As such, results from the study indicate that the majority of users did not disclose gender, and thus the gendered frequencies are much lower than the total frequencies. Finally, YouTube data was likely underestimated, as Brandwatch only captured brand posts in text, such as in YouTube comments and captions, not in any images or videos. It is also important to note that this study did not analyze the nutritional quality of the food and beverage products that were featured in the posts, and it is possible that some of the discussed posts and reach were for products that more healthful food offerings from the included brands.

## 5.0 Conclusion

This novel study benchmarked posts featuring popular food and beverage brands on various social media sites. The results from this study clearly indicate that social media is a part of the food environment that needs to be monitored by governments. Given that children and adolescents are present on social media sites and are vulnerable to unhealthy food marketing as well as to peer influence, policies designed to protect children’s health must absolutely consider the digital food environment which is likely contributing to the normalization of unhealthy food and beverage intake. Potential policies could include educational campaigns for both parents and children about the impact of digital marketing and social media marketing campaigns encouraging parents to develop stricter controls on their children’s social media use however, such approaches would likely have limited impact and reach. Upstream policy approaches such as mandatory government regulations that push social media companies to identify and limit exposure to unhealthy food marketing viewed by children need to be considered in order to protect children’s health.

## Supporting information

S1 Appendix Data Coverage on Brandwatch. (DOCX)
