# Supplementary material for: Normalizing junk food: The frequency and reach of posts related to food and beverage brands on social media
Source: PLOS Digit Health. 2024 Oct 31;3(10):e0000630. doi: 10.1371/journal.pdig.0000630 (PMC11527147; doi:10.1371/journal.pdig.0000630)
Supplement: S1 Appendix — (DOCX) [file pdig.0000630.s001.docx]

**S1 Appendix**. Data Coverage on Brandwatch

| Social Media Platform | Brandwatch Coverage |
| --- | --- |
| Twitter | - - - Brandwatch is an official partner with Twitter, so it has complete access to all Twitter data, including historical and real-time data. |
| Facebook | - - - Brandwatch can only collect Facebook data through channels, which are specific social media profiles that you add to Brandwatch which give you access to posts, comments, and other metrics on any given profile     - Limited historical backfill |
| Instagram | - - - Like Facebook, Instagram can only be tracked via channels, and has limited historical backfill. |
| YouTube | - - - Brandwatch collects YouTube data via third party data providers.     - Data is collected from text descriptions of videos, comments under videos, and metadata. |
| Reddit | - - - Brandwatch has full access to all active subreddits, with strong historical and real-time data coverage |
| Tumblr | - - - Full access to an unfiltered, real-time stream of Tumblr data, with over 100 million mentions spanning back to December 2014. |

Source: Brandwatch 2021
